# Online Health Information Seeking Using “#COVID-19 Patient Seeking Help” on Weibo in Wuhan, China: Descriptive Study

**DOI:** 10.2196/22910

**Published:** 2020-10-15

**Authors:** Xiaoman Zhao, Ju Fan, Iccha Basnyat, Baijing Hu

**Affiliations:** 1 Research Center of Journalism and Social Development Renmin University of China Beijing China; 2 School of Journalism and Communication Renmin University of China Beijing China; 3 Data Engineering and Knowledge Engineering Lab & School of Information Renmin University of China Beijing China; 4 School of Communication Studies James Madison University Harrisonburg, VA United States

**Keywords:** COVID-19, coronavirus, information seeking, social media, Wuhan

## Abstract

**Background:**

First detected in Wuhan, China in December 2019, the COVID-19 pandemic stretched the medical system in Wuhan and posed a challenge to the state’s risk communication efforts. Timely access to quality health care information during outbreaks of infectious diseases can be effective to curtail the spread of disease and feelings of anxiety. Although existing studies have extended our knowledge about online health information–seeking behavior, processes, and motivations, rarely have the findings been applied to an outbreak. Moreover, there is relatively little recent research on how people in China are using the internet for seeking health information during a pandemic.

**Objective:**

The aim of this study is to explore how people in China are using the internet for seeking health information during a pandemic. Drawing on previous research of online health information seeking, this study asks the following research questions: how was the “#COVID-19 Patient Seeking Help” hashtag being used by patients in Wuhan seeking health information on Weibo at the peak of the outbreak? and what kinds of health information were patients in Wuhan seeking on Weibo at the peak of the outbreak?

**Methods:**

Using entity identification and textual analysis on 10,908 posts on Weibo, we identified 1496 patients with COVID-19 using “#COVID-19 Patient Seeking Help” and explored their online health information–seeking behavior.

**Results:**

The curve of the hashtag posting provided a dynamic picture of public attention to the COVID-19 pandemic. Many patients faced difficulties accessing offline health care services. In general, our findings confirmed that the internet is used by the Chinese public as an important source of health information. The lockdown policy was found to cut off the patients’ social support network, preventing them from seeking help from family members. The ability to seek information and help online, especially for those with young children or older adult members during the pandemic. A high proportion of female users were seeking health information and help for their parents or for older adults at home. The most searched information included accessing medical treatment, managing self-quarantine, and offline to online support.

**Conclusions:**

Overall, the findings contribute to our understanding of health information–seeking behaviors during an outbreak and highlight the importance of paying attention to the information needs of vulnerable groups and the role social media may play.

## Introduction

### Background

On March 11, 2020, the World Health Organization (WHO) declared the COVID-19 outbreak a pandemic [1]. First detected in Wuhan, China in December 2019, the disease rapidly spread into more than 100 locations internationally, including Japan, Korea, and the United States [1]. In the Report of the WHO-China Joint Mission on COVID-19, the Joint Mission reminded the public that the virus is unique in its ability to cause societal and economic disruption [2]. In China, the disease has caused 3176 deaths out of the 80,813 total confirmed cases by March 12, 2020 [3]. The rapid increase in coronavirus patients stretched the medical system in Wuhan, which accounts for 60% of mainland China’s total confirmed cases and about 77% of the deaths [4]. In an attempt to limit the spread of the virus, Chinese government has enforced an unprecedented lockdown on Wuhan by suspending transport both within the city and leaving the city [5]. During the quarantine, each household was allowed to have only one person go out for necessities every 2 days [6]. The traffic ban within the city has made it hard for patients to seek health care [7]. It was also difficult to organize medical care, frequently monitor disease progression, and refer hospital care for patients in home isolation in a timely manner [8]. The lockdowns caused public panic and resulted in many cross-infections at the crowded, overwhelmed hospitals [9], posing a challenge to the state’s risk communication efforts. The dramatic increase in the number of infected individuals was causing a burden on the medical system [10].

Timely access to quality health care information during outbreaks of infectious diseases can be effective to curtail the spread of disease and feelings of anxiety [11,12]. Up-to-date information about specific threats and necessary precautionary measures was found to mitigate public anxiety, reduce morbidity and mortality, and contribute to minimizing negative mental impacts [13,14]. Furthermore, previous studies relate health anxiety, internet literacy, and chronic conditions to the willingness of individuals to engage in health information–seeking behaviors [15-17]. Health information–seeking behavior is purposeful activities such as searching for condition-specific information as well as disease prevention and treatment information to fulfill specific health information need [18,19]. An information need can arise when a patient experiences health-related uncertainty originating from an inaccurate, inconsistent, ambiguous, or excessive provision of information about the diagnosis, treatment, or aspects of medical decision making [16,20,21]. However, relatively little is known about what happens to the information-seeking behavior of patients during an outbreak, especially in the context of China. Therefore, this study explores the information-seeking behaviors during the coronavirus outbreak in China.

The internet, especially social media, has also been identified as a significant source for information searching and decision making [22]. Scholars suggest potential in the internet to supplement traditional sources of health information and to support patients’ decision making [22,23]. In fact, health information seeking has been found to be a popular online activity [24]. Thus, researchers have suggested that an analysis of web behaviors can provide insights into individuals’ information seeking during an outbreak, as public reactions are visible more quickly online [12,25]. For instance, by monitoring web activities, previous studies identified large increases in activities on social networking websites, including posting and searching, that are closely associated with the outbreaks of pandemic diseases [26,27]. Text analysis programs that are developed for measuring emotional expression in natural language are also found to produce reliable results that are congruent with human ratings [28,29]. Using web-based text analysis to monitor public emotions is also suggested by researchers to avoid self-report biases from social desirability effects or memory distortions [12]. Therefore, using entity identification and text analysis on a total of 10,908 posts on Weibo, the largest social networking platform in China, we identified 1496 patients with COVID-19 living in or with family in Wuhan, China and explored their online health information–seeking behavior during a pandemic.

### Online Health Information Seeking

Health information seeking has been found to be a popular online activity. Studies in the United States and Europe have reported more than 70% of internet users have looked online for health information of one kind or another [24], or having used the internet for health purposes [30]. The motivations for seeking online health information are diverse, including self-diagnosing, coping with uncertainty, staying informed on preventing diseases, and looking for others with a similar health concern [24,31]. Through query construction and information source selection, information seekers can enjoy greater control over information acquisition processes and achieve desired levels of uncertainty [32]. In view of the features such as convenience, cost effectiveness, and private sharing, scholars suggest potential in the internet to supplement traditional sources of health information and support patients’ decision making [22,23].

Previous studies have identified multiple factors that may influence patients’ motivation to seek health information online, including biological sex, income, age, chronic illness, and travel time to offline sources of health care [16,17,33,34]. For instance, biological sex was found to significantly predict online health information seeking, with females more likely to seek out online health information than males—perhaps because females often take on primary caregiving roles in families and are more cautious in risk contexts [16,35]. Increased age is frequently associated with decreased levels of motivation for health-related information seeking online, as older adults are always found to have lower levels of internet literacy and experience more difficulties navigating websites [36,37]. Further, the digital divide, a gap between individuals from different socioeconomic backgrounds with regard to their access to and use of digital equipment and services, can increase challenges associated with online health information seeking, such as inequality of accessibility and difficulties to differentiate between high and low quality resources [17,21].

Additional factors, such as efficacious feelings about using the internet and health anxiety, may also influence one’s motivation of seeking out health information online [16,34,38]. User experience online was also found to influence one’s feelings of efficacy and, thus, be linked to their likelihood to use online search strategies for health-related information [34]. Adding on to self-efficacy and health anxiety is chronic illness, as individuals with a chronic illness were more likely to use the internet to search for health information compared to those without a chronic illness [39]. A long travel time to offline sources of health care was also associated with a stronger likelihood of using the internet to find health information [17].

Although these existing studies have extended our knowledge about online health information–seeking behavior, processes, and motivations, rarely have the findings been applied to an outbreak. Therefore, in this study, we examine the ways in which patients with COVID-19 and families living in Wuhan, China used the internet to seek health information on the social medial platform Weibo. The findings can help build an understanding of how the internet can be used to better serve the needs of the public, especially the patients at the peak of the outbreak, given the high level of uncertainty and risks.

### Online Health Information Seeking in China

In China, people are found to face challenges in accessing health care resources, among which are the difficulty in making medical appointments, short consultation times, and a significant socioeconomic disparity in health literacy [40,41]. Despite the governmental attempt to reform its health care system, patients still expressed unsatisfied needs for various kinds of health-related information, including the treatment of diseases and the effect, etiology, and risk factors, as well as use, of drugs and medication [42]. The barriers to accessing accurate information and subsequent health care also lead to people’s lack of trust in doctors and their unwillingness to visit them [42]. In this context, the internet has been increasingly used to access health information, supplementing the traditional sources of health information [43,44].

According to statistics from the government-run China Internet Network Information Centre, the number of internet users in the country had skyrocketed to 854 million at the end of June 2019, with the internet penetration rate reaching 61.2% [45]. Although 18.2% of the total Chinese population are 60 years or older [46], users older than 60 years accounted for only 6.9% of all users on the internet [45]. A survey study found that 36.7% of the Chinese participants had sought online health information at least one or two times, citing saving money and easing the privacy concern as the two major reasons for seeking out health information online [43]. Despite the increase in health-related internet use in China, there is relatively little recent research on how people in China are using the internet for seeking health information. Almost all the studies were limited to individuals that were young and educated with certain levels of online health information–seeking experience, mostly in Hong Kong [42-44,47]. According to these studies, the overall health literacy level in China is lower than that in Western countries [48].

The majority of online health information was found to be of poor quality, and the functions of health websites were ineffective and hard to navigate [43]. However, information seekers in China have still been found to consider the internet a highly reliable source of information [49]. Although some studies reported no correlations between education or gender on Chinese patients’ online health information–seeking behaviors [42,50], some other studies have identified digital inequalities associated with education level, household income, and socioeconomic status, further leading to variations in personal health condition and family well-being [44,51]. These disagreements in the findings point to the need to conduct further research on how people in China, especially the general population in mainland China, are using the internet for health information, particularly during a pandemic. Therefore, drawing on previous research of online health information seeking, this study asks the following research questions: How was the “#COVID-19 Patient Seeking Help” hashtag being used by patients in Wuhan seeking health information on Weibo at the peak of the outbreak? What kinds of health information were patients in Wuhan seeking on Weibo at the peak of the outbreak?

## Methods

### Data Collection

During the COVID-19 outbreak, Weibo, the largest social networking platform in China, created a hashtag named #*COVID-19 Patient Seeking Help* (“*Feiyan Huanzhe Qiuzhu Chaohua*”) for the patients and their families to leave their name, age, city, neighborhood, address, time of sickness, health condition, additional description, and contact information, making the posts structured. For the purpose of identity verification, patients were also asked to upload pictures of a medical examination, if any, which further improved the credibility of the data. We crawled and analyzed Weibo posts with this hashtag published from January 29, 2020, when the hashtag was first created, to February 17, 2020, to examine the online health information–seeking behaviors of patients with COVID-19. This period of 20 days was chosen because, by the end of this period, the number of patients posting with this hashtag fell to zero. In total, 10,908 Weibo post entries with the #COVID-19 Patient Seeking Help hashtag were collected.

For each post, in addition to the structured patient information previously noted, we further extracted the following items: *the date and time of posting, user ID, user gender, and URL of the thread* (for further referring back to the entry online). We excluded retweets and general comments about the outbreak. After this step, we obtained 4983 entries of patients with COVID-19 in Wuhan using the hashtag *#COVID-19 Patient Seeking Help*. Data consolidation was further carried out by patient name and detailed address, which resulted in 1496 unique patient cases.

We further standardized the patients’ information that was crawled, as the language used on the social media platform was flexible. In particular, to standardize the patients’ detailed address, we crawled a full list of housing estates in Wuhan, from the largest housing estates website in China, Lianjia [52], and mapped the original Weibo texts with patient’s address to the housing estate names. We further obtained the longitudes and latitudes of the patients’ locations through the Baidu map application programming interface (API) [53].

### Data Analysis

Previous studies identified multiple factors that may influence patients’ motivation to seek out health information online, including biological sex, age, chronic illness, and travel time to offline sources of health care [16,17,33,34]. To answer the first research question, we examined the age of the patients. The original posts did not include patient gender, but we examined the gender of the posting users, as previous studies have found that about half of individuals’ health information searches are on behalf of someone else’s health situation [24,54]. We also examined the patients’ underlying condition by extracting the health condition description in each entry. Specifically, we conducted word segmentation using the Chinese word segmentation module, Jieba [55]; computed document frequency for every single term that appeared in the content; and identified the terms indicating their underlying diseases.

We also examined the patients’ shortest walking distance to offline sources of health care. Specifically, we extracted the list of 42 fever clinics and the 28 designated hospitals that was first published by the Health Commission of Hubei Province [56] and further updated by the Hubei Provincial People’s Government [57]. Through Baidu map API, we further obtained the longitudes and latitudes of these clinics and hospitals, and calculated the shortest walking distance of each patient’s location to the nearest fever clinic or designated hospital, as public transportation and private car driving in the city were prohibited during the period of our study, and patients may have faced difficulties seeking medical care due to the traffic ban [7].

To answer our second research question, we examined their information-seeking behavior, as indicated by the number of entries posted by each patient or user. We also examined the specific information they were seeking out by analyzing the content of health condition and additional description in each entry, where the patients had given more details about their needs. We carried out textual analysis on every post to identify the information needs of the patients. Specifically, thematic analysis was carried out. Open coding, the first step of the coding process entailed reading each entry and its messages, highlighting salient phrases and words [58,59]. At this stage, one of the authors conducted the open coding, reading the entries in Chinese. More than 200 open codes were generated in this process. Some examples of open codes that emerged were queuing for test, called every hospital for bed, no foreseeable treatment, staying overnight for injection, the hospital is full of patients, waiting for confirmatory testing result, rushing between different hospitals, hundreds of people in the waiting list, reported to the neighborhood committee with no response, and have to find solutions by ourselves. Next, the open codes and phrases were conceptually clustered into more than 30 different axial code groupings through discussion among the authors [59]. Keeping the research question in mind, through additional discussion and data refinement, the authors agreed that the following themes best answered the research question: accessing medical treatment, managing self-quarantine, and accessing tangible support.

## Results

**Figure 1:**
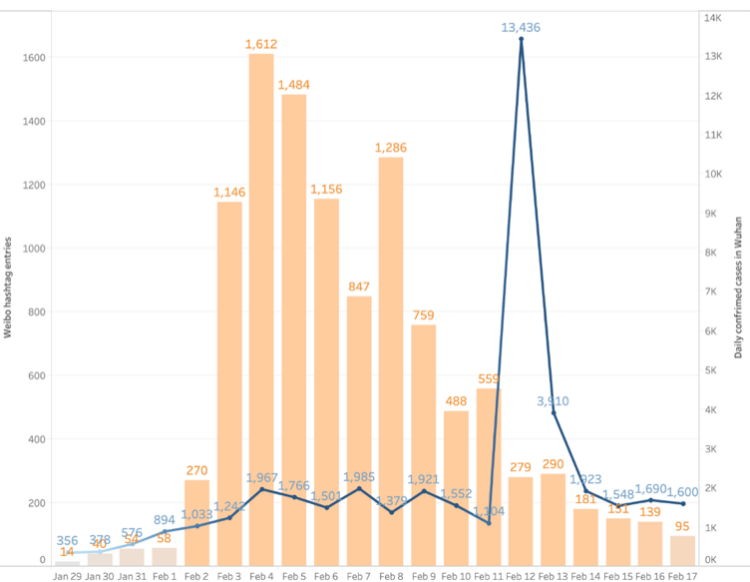
Daily numbers of #COVID-19 Patient Seeking Help hashtag entries (the orange bar plot) and daily confirmed cases in Wuhan (the blue line plot).

Figure 1 presents the numbers of Weibo posts with the #COVID-19 Patient Seeking Help hashtag and the daily number of confirmed cases in Wuhan reported by the Health Commission of Hubei Province. Figure 1 illustrates that the number of entries rapidly grew from February 3, 2020, and maintained a high level until February 12. On February 12, the daily confirmed cases peaked at 13,436, at which time the central government promised to admit all the patients with COVID-19 [60]. The number of the hashtag entries have steadily declined since then.

Table 1 is an age comparison between our sample and that of the WHO-China Joint Mission [2]. The WHO-China Joint Mission identified the median age of patients as 51 years based on a total of 55,924 confirmed cases. However, for our sample, the median age was 61 (IQR 50-70) years, with an average age of 59 years. According to the report published by the WHO-China Joint Mission, individuals 60 years or older are at highest risk for severe disease and death. Previous studies have also shown an age-related digital divide in China. Although 18.2% of the total Chinese population were 60 years or older by the end of 2019 [46], users older than 60 years accounted for only 6.9% of all users of the internet [45]. The age-related digital divide might prevent the patients from seeking information and help online.

**Table 1:** Age comparison between our sample and that of the WHO-China Joint Mission.^a^

Sample	Participants, n	Age range	Age IQR (years)	Age median (years)
WHO^b^-China Joint Mission	55,924	2 days-100 years	39-63	51
Our sample	1454	2-99 years	50-70	61

^a^Age was missing in 42 Weibo entries.

^b^WHO: World Health Organization.

Table 2 shows the document frequency of family members’ names that appeared in the posts. Out of 883 entries, words such as “mum (mother),” “dad (father),” and “elder at home” appeared in 35% (n=308), 29% (n=255), and 24% (n=209) of the entries, respectively. Further examination of these entries showed that most of the entries were posted by the younger generation for their parents or an older adult at home. We examined the gender of the users seeking out health information. Among the 2405 unique users, 69% (n=1660) were female and only 31% (n=745) were male. The median number of posting times by female users was 2 (IQR 1-2) times, which was higher than that of male users, 1 (IQR 1-2) time. It shows that there were more female users than male users trying to seek out health information online, and their internet use frequency might be higher than their male counterpart.

**Table 2:** Document frequency of names of family members.

Word	Frequency (n=883), n (%)	Quote
Mother (mum)	308 (35)	“My *mother* is highly suspected with COVID-19. She could not even get up right now...She has been running a fever for more than ten days...”
Father (dad)	255 (29)	“I am the son of the patient. My *father* has been infected with pneumonia. And the lung lesions are quite serious. I have reported to the neighborhood committee for many days, but they have not arranged hospital bed for us...”
Elder at home	209 (24)	“The *elder at home* have been diagnosed with the pneumonia...Please contact his daughter as the *elder at home* do not use the Internet.”
Grandma	88 (10)	“Now my *grandma* is already in incontinence, but the neighborhood committee still asks us to wait.”
Parents	80 (9)	“My *parents* are both confirmed and in dangerous condition. But we haven’t received hospitalization notification. Please save my *parents*.”
Grandpa	62 (7)	“The patient is my *grandpa*, who is in dangerous condition. He has emphysema and threatening myocardial infarction.”
Aunt	57 (6)	“The *aunt* is currently in recurring fever. We are all in desperation.”
Uncle	52 (6)	“My *uncle*’s condition is worsening in self-quarantine, with eating and breath difficulties.”

**Figure 2:**
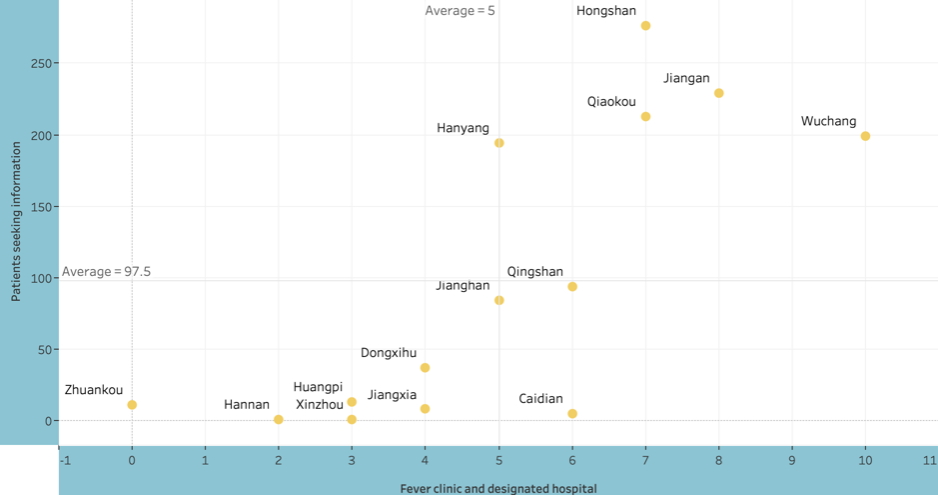
Number of patients seeking health information and number of fever clinic and designated hospital by district (yellow point).

We further examined the patients’ distance from their residential locations to offline health care. We first extracted the district of each patient’s residential location to see the distribution of these patients across different districts of Wuhan. Figure 2 shows the number of patients in our sample by district. Districts of Hongshan, Jiangan, and Qiaokou were found to have the most patients seeking information online.

**Figure 3:**
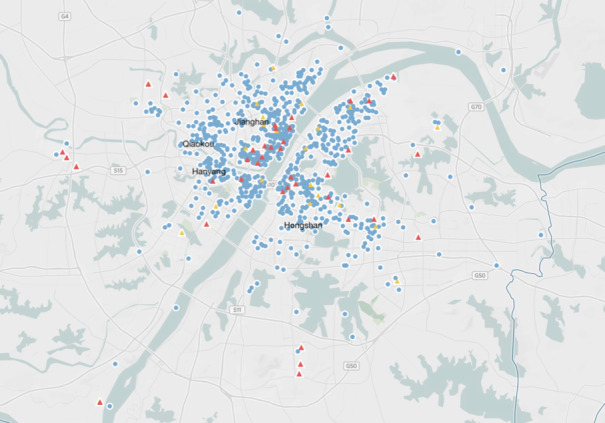
Overview of the locations of patient residential addresses (blue points), fever clinics (red triangle), and designated hospitals (yellow triangle).

We also examined the geographic distribution of the fever clinics and designated hospitals in relation with the patients’ residential locations. Figure 3 is an overview of all the patients’ locations, the fever clinics, and the designated hospitals on a map of Wuhan City. Seen from the map, in the districts of Hongshan, Jiangan, and Qiaokou, some patients were far from the fever clinics and designated hospitals.

To further examine the exact distance of the patients to offline health care resources, we computed the distance between every residential location to its nearest fever clinic or designated hospital. Table 3 maps the patients’ distance by district to the nearest fever clinic or designated hospital, and the average distance was 2.67 (SD 2.88) km. According to estimation by the Baidu map API, it takes 50 minutes to walk 3 km by an adult. Considering the average age of our sample was older, their actual walking time could be even longer. This means that 27.6% (n=413) of the sample had to walk for around 1 hour to access offline health care. In the context of our study, distance to offline heath care is especially important. Due to the transport prohibition and quarantine enforced, patients could only walk to access health care offline. The difficulties to travel were also cited by many users as a major reason for going online to seek health information as identified in textual analysis.

**Table 3:** Patients’ distance to the nearest fever clinic or designated hospital.

District	<1 km distance, n	1-2 km distance, n	2-3 km distance, n	>3 km distance, n
Qiokou	68	17	4	124
Hongshan	28	66	67	116
Jiangan	68	58	35	68
Hanyang	28	77	52	38
Dongxihu	3	0	7	27
Jianghan	36	22	11	15
Wuchang	64	91	38	6
Huangpi	5	3	0	5
Zhuankou	0	6	1	4
Jiangxia	0	0	2	4
Caidian	0	2	0	3
Xinzhou	0	0	0	1
Qingshan	39	39	15	1
Hannan	0	0	0	1
All districts	339	381	232	413

Table 4 shows the patients’ underlying conditions, which were extracted from the health condition description in each entry. Document frequency analysis shows that “*hypertension*,” “*diabetes*,” “*heart disease*,” and “*underlying disease*” were mentioned in 12% (n=110), 9% (n=82), 9% (n=79), and 3% (n=30) of the 883 posts, respectively. According to the report published by the WHO-China Joint Mission, individuals with underlying conditions such as hypertension and diabetes are at highest risk for severe disease and death [2].

**Table 4:** Document frequency of terms indicating patients’ underlying condition.^a^

Word	Frequency (n=883), n (%)	Quote
Hypertension	110 (12)	“The patient has *hypertension*, diabetes and other underlying conditions. He has had diarrhea for five days with continuous wheezing and breathing difficulties...”
Diabetes	82 (9)	“My father has multiple underlying diseases, including *diabetes* and hypertension. The CT^b^ scan shows ground glass opacity. We have reported to the community hospital and they said that they could do nothing...”
Heart disease	79 (9)	“My grandpa has a history of *heart disease* for years and received emergency treatment for several times before.”
Underlying disease	30 (3)	“The CT scan shows ground glass opacity in both lungs. My father has serious *underlying disease* of cardiomegaly. He needs to be hospitalized immediately...”

^a^Health condition was missing in 613 Weibo entries.

^b^CT: computed tomography.

**Figure 4:**
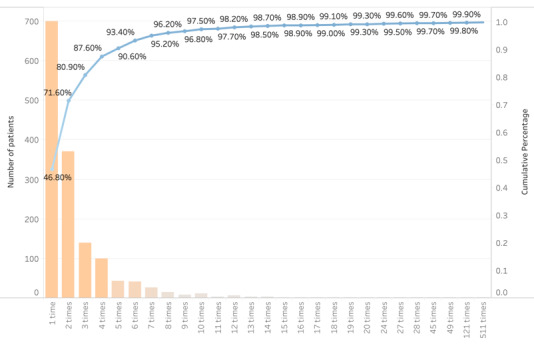
Posting times per patient.

Our second research question asks what kinds of health information patients in Wuhan were seeking on Weibo at the peak of the outbreak. We first examined the frequency of posting per patient. Seen from Figure 4, most of the patients have posted at least 3 times, which accounted for 80.9% (n=1211) of the sample. Still, another 14.2% (n=213) of the sample posted 4-7 times. Although some patients did post more than 7 times, it was rare, which accounted for only 4.8% (n=72) of the sample.

We also examined the specific information that these patients or their families were seeking. Textual analysis showed that information about accessing medical treatment, especially hospital beds and confirmatory testing, was most sought out by the patients. In those posts, the users shared their experience of “*running around for medical treatment*,” “*being denied service and/or a bed*,” and for “*not knowing how to proceed*.” Turning to the hashtag, posters were desperately seeking information on health care. Feelings of desperation and loss of desire for survival were prevalent, such as “*my mother is very desperate, telling me no need for treatment any more. She has lost confidence to survive*” and “*my mom’s desire for survival is waning*.”

The posters also sought information about managing self-quarantine. Due to the shortage of offline health care, the neighborhood committees and hospitals usually suggested the patients to stay at home to conduct self-quarantine but did not help patients make strategic decisions about self-treatment. The posting highlighted that the patient’s “*illness deteriorated*” due to “*a lack of proper treatment and nutritious food*.” Many users recounted experience of “*infection among family members*” living under the same roof.

In addition to information and advice, the patients were also requesting for possible ways to provide tangible resources such as “*childcare*” and “*transportation to seek offline health care*.” Particularly, many requested for social support to “*check in on elderly parents*” living in Wuhan, to “*take them to the hospital*,” or to “*help them with their medication*,” as they were isolated in a different province, different city, or different district. Although the lockdown policy cut off their offline social support network, the online platform provided a possibility to seek help.

Overall, the social media platform played a vital role connecting health information seekers with a reliable and timely source of information, tangible support, as well as a more empathetic crowd. This source provided not only informal advices but also important, timely discussion and caring interactions. Posts such as “*I could do nothing other than following the updates on Weibo everyday*,” “*thank all the kind-hearted people sending me clues and suggestions. Thank you for your empathy*,” and “*my grandpa has been hospitalized. Hope every patient seeking help could be as lucky as me. Thank you all for your kind help*” highlight the intangible benefits of using the online platform during the pandemic to seek health information.

## Discussion

### Principal Findings

In this study, we examined how patients with COVID-19 living in Wuhan, China used the “*#COVID-19 Patient Seeking Help*” hashtag to seek health information. Our findings provide important insights into health information–seeking behaviors during pandemic outbreaks. The curve of the hashtag posting provided a dynamic picture of public attention to the COVID-19 pandemic. Previous studies suggest that an analysis of web behaviors can provide insights into individuals’ information seeking during an outbreak, as public reactions are visible more quickly online [12,25]. In our study, we identified a rapid increase in posting under the #COVID-19 Patient Seeking Help hashtag at the onset of the pandemic outbreak and a decrease following the government’s effort to admit every patient. The steep curve of the hashtag indicates that online information–seeking behaviors such as posting, commenting, and reposting are useful markers of public reaction and draws attention to the need for public health practitioners to pay attention to online space in their responses. This finding is consistent with research identifying an increase in activities on social networking websites following the outbreaks of pandemic diseases [26,27].

In general, our findings confirmed that the internet is used by the Chinese public as an important source of health information. Previous studies have associated increased age with decreased levels of motivation for health-related information seeking online [36,37]. Similarly, our findings highlighted that younger family members primarily sought information online for parents or for older adult patients at home [61]. Furthermore, our findings highlight the ability to seek information and help online, especially for those with young children or older adult members during the pandemic. This finding deserves consideration in the context of China, considering the age-related digital divide and the decline in health information–searching behaviors among older adults [50]. Although timely access to quality health care information during outbreaks is vital for reducing morbidity and mortality [13,14], it is equally important to pay attention to group-specific health information needs and their ability to act upon the information.

In our sample, we also identified a high proportion of female information seekers, which was consistent with previous studies that found females more likely to seek online health information [16,35]. However, posting frequency was comparable between female and male seekers in our sample. Another factor that may have contributed to the patients’ use of the internet for health information is the long travel time to access offline health care resources. Previous studies indicated that the cost associated with time to visit health care providers in traditional settings has influence on patients’ motivation for seeking out health information on the internet [17]. In our sample, around 30% of the patients with COVID-19 lived in a distance more than 3 km from their nearest clinic or designated hospital. The suspension of transportation in Wuhan meant that patients had to walk for at least an hour one way to access an offline health care source. The difficulties in travelling to clinics or hospitals were also cited by many as a major reason to seek health information and help from online platforms. The lockdown policy was also found to cut off the patients’ social support network, preventing them from seeking help from family members. Social support was sought on social media to check in on older adults, to take them to the hospital, or to help them with medication, which highlights the vulnerability of this population despite the effectiveness of the policy in containing the disease.

Our findings give insight into the issues that patients and their families were most concerned about during the peak of the outbreak, including where and how to seek medical treatment and confirmatory testing, decision making on self-quarantine, and experience of infection among family members. Previous work indicated that an information need can arise when a patient experiences health-related uncertainty and, in turn, engages in health information–seeking behavior to get reassurance, to manage uncertainty, and to reconcile oneself with a new health situation [16,21]. Our findings highlight a need for information originating from the stretched condition in the health care system and the anxiety over the lack of access to proper treatment. To the patients and their families, the act of searching for information online is a help-seeking step so that they can manage their own health with the affordance of the internet. Scholars suggest this kind of behavior should be encouraged as an integral and positive part of the patients’ journey because online health information seeking enables patients to accumulate more social support, which is associated with better health outcomes and heath decision making [17,48].

Methodologically, our study also indicates the usefulness of using a computational method to explore individuals’ responses to public health crises in real time. For example, the increase and then decrease in public anxiety eased by the communication effort in response to the H1N1 epidemic was hard to capture by traditional survey methods [12]. Consistent with previous studies, our study shows that the number of entries with the #COVID-19 Patient Seeking Help hashtag rapidly grew and was kept at a high level within a period of more than 1 week and then steadily declined following the government’s effort to admit every patient [60]. By monitoring and analyzing the patients’ online data, our method enables a possible advantage over traditional approaches to offer a dynamic picture of changes in public response to the pandemic in real time.

Our study also helps build an understanding of how the internet can be used to better serve the needs of the public, especially the patients in the time of an outbreak. In general, our findings confirmed that the internet is used by the Chinese public as an important source of information and help. Although previous studies mainly focused on the online health information–seeking experience of the young and educated [43,44,47,51], our findings highlight the needs of older adults, who may have equal motivations but lack the ability for searching and comprehending online health-related information. Therefore, in addition to making relevant and high-quality information available online, it is vital to motivate social support to facilitate their information needs.

### Limitations and Conclusion

Using a nonprobability and convenience sample, this study focused on basic descriptive analyses of how people in China are using the internet for seeking health information during a pandemic. Nonprobability sampling means there lacks a sound theoretical basis for statistical inference [62]. Future studies using random sampling are needed to allow valid statistical analysis so that informed judgments can be made. Another limitation of our study is the lack of our ability to establish direct links with the patients’ health outcomes. More in-depth discussion is needed to explore whether and how the information-seeking behaviors on social media aids in better health outcomes. Future studies should explore the link between information need and patients’ health outcomes.

The COVID-19 pandemic has been found to stretch the local medical system and poses a challenge to the state’s risk communication efforts. Social media is used by the patients to seek health information relevant to the outbreak. Some factors may contribute to their online information-seeking motivation including age, gender, underlying conditions, and travel time to offline health care service providers. Overall, the findings contribute to our understanding of health information–seeking behaviors during an outbreak and highlight the importance of paying attention to the information need of vulnerable groups and the role social media may play.

## Abbreviations

API: application programming interface
WHO: World Health Organization
